# Identifying impaired mental health in patients with type 2 diabetes: a cross-sectional study in general practice

**DOI:** 10.3399/BJGPO.2024.0045

**Published:** 2025-01-29

**Authors:** Line T Jakobsen, Anne Søjbjerg, Stinne E Rasmussen, Kaj S Christensen

**Affiliations:** 1 Research Unit for General Practice, Aarhus University, Aarhus, Denmark; 2 Department of Public Health, Aarhus University, Aarhus, Denmark

**Keywords:** mental health, screening, diabetes, diabetes mellitus, general practice

## Abstract

**Background:**

Type two diabetes (T2D) is linked to impaired mental health. International guidelines emphasise the importance of including psychological aspects in diabetes care. Yet, no systematic approach has been implemented to assess mental health in patients with T2D in general practice.

**Aim:**

To evaluate the mental health of patients with T2D in general practice, and to investigate the effectiveness of asking patients about their wellbeing by using a single-item question compared with the five-item World Health Organization-Five Wellbeing Index (WHO-5).

**Design & setting:**

A cross-sectional study was undertaken, which included 230 patients with T2D in Danish general practice, from 1 May 2023–31 January 2024.

**Method:**

Eligible patients were recruited at the annual chronic care consultation. They answered a single-item question on wellbeing and four validated measures of general wellbeing (WHO-5), depression (Patient Health Questionnaire-9; PHQ-9), anxiety (Generalised Anxiety Disorder-7; GAD-7), and diabetes distress (Problem Areas in Diabetes-5; PAID-5).

**Results:**

Overall, 32% of patients expressed symptoms of impaired mental health. Notably, the WHO-5 identified 53% of these patients, whereas only 12% of patients were identified through the single-item question. Importantly, among the patients exhibiting symptoms of impaired mental health, those identified by the WHO-5 displayed statistically significantly lower mental health scores across all measures (except PAID-5) compared with those not identified by the WHO-5.

**Conclusion:**

A significant proportion of patients with T2D in general practice are affected by mental health issues. Our findings indicate that a single-item question may not sufficiently detect these issues, highlighting the importance of incorporating tools, such as the WHO-5, to offer a more comprehensive approach in diabetes care.

## How this fits in

Impaired mental health is common in patients with type two diabetes (T2D). Consequently, routine care should include a regular assessment of mental health. However, there is a lack of research on assessing mental health in patients with T2D in general practice. This study highlights the need to prioritise mental health in this population in this setting, demonstrating the effectiveness of the World Health Organization-Five Wellbeing Index (WHO-5) as a systematic screening tool.

## Introduction

The prevalence of T2D is increasing. Projections anticipate the worldwide prevalence of T2D to reach 1.3 billion by 2050.^1^ Patients with T2D are typically treated in primary care, particularly in general practice, with scheduled annual chronic care consultations.

It is well established that impaired mental health is common in patients with diabetes, as one in five patients are affected.^2–6^ However, previous research often samples patients with both type one and type two diabetes without a specific emphasis on general practice. Consequently, there is a gap in our understanding of the mental health status in individuals with T2D from a general practice perspective. Recognising signs of mental health issues in patients with T2D is crucial; they do not only impose a psychological burden, but they are also associated with poorer diabetes treatment outcomes.^7–10^ Furthermore, improvement in depressive symptoms is linked with improved glycaemic control.^11,12^ Hence, routine management of these patients should assess both physical and mental health, thereby laying the foundation for effective interventions aimed at enhancing mental health in this population.

Numerous national T2D guidelines emphasise the importance of addressing mental health.^13–16^ Furthermore, the International Diabetes Federation (IDF) recommends periodic, regular assessments of mental health, either through direct questioning or validated measures, such as the WHO-5.^17–19^ Despite these recommendations, reliable assessment tools are not widely used to systematically examine mental health in patients with T2D.^20^ Consequently, additional research is called for to identify the most effective approaches in this area.

The aim of this study was twofold. First, we aimed to explore the mental health of patients with T2D in general practice. Second, this study aimed to evaluate the suitability of the WHO-5 as a screening instrument for mental health in comparison with other commonly used tools, and to assess its effectiveness against a single-item question for determining mental health status.

## Method

### Design

This cross-sectional study was based on a questionnaire survey administered to patients with T2D treated in general practice.

### Setting

The study was conducted in 12 Danish general practices from 1 May 2023–31 January 2024. The Danish healthcare system is mainly tax-funded, and all residents have free and equal access to most services, including general practice. Patients with T2D treated in general practice are offered at least one annual chronic care assessment, which comprises two consultations: one for paraclinical tests and another for evaluating test results, adjusting medication, and updating the treatment plan.^21^

### Recruitment

#### Recruitment of clinics

General practice clinics were recruited via email as a convenience sample. Out of 35 clinics recruited, 12 agreed to participate. Six of the clinics were located in urban areas and six in rural areas, ensuring a diverse population for the study.

#### Recruitment of patients

The healthcare professionals were directed to consecutively enrol patients diagnosed with T2D during the annual assessment for chronic care. The patients received oral and written information about the study from their healthcare professional before providing oral consent to participate. In Denmark, written consent is not required for questionnaire-based studies.^22^

The inclusion criteria were patients aged ≥18 years, diagnosed with T2D, and treated in general practice. The exclusion criteria were individuals unable to read and speak Danish.

### Questionnaire

The questionnaire comprised five different patient-reported outcome measures (PROMs): a single-item question on wellbeing ('How is your wellbeing at the moment?') and four validated psychometric measures widely used internationally (Table 1). The single-item question aimed to imitate the current standard approach for assessing the patient’s wellbeing and had five response options: very good, good, neutral, bad, or very bad. The psychometric measures included the WHO-5 questionnaire, the Patient Health Questionnaire-9 (PHQ-9),^23^ the Problem Areas in Diabetes-5 (PAID-5),^24^ and the Generalised Anxiety Disorder-7 (GAD-7).^25^

**Table 1.** Overview of patient-reported outcome measures

Mental health measure	Items, *n*	Score range	Cut-off value	Topic
Single-item wellbeing question^a^	1	1–5	≥4: impaired mental wellbeing	Mental wellbeing
WHO-5 Wellbeing Index^b^ ^18^	5	0–100	<50: impaired mental wellbeing	Mental wellbeing
Patient Health Questionnaire-9^c^ ^22^	9	0–27	0–4: no depressive symptoms 5–9: mild depressive symptoms 10–14: moderate depressive symptoms 15–19: moderately severe depressive symptoms ≥20: severe depressive symptoms	Depressive symptoms
Problem Areas in Diabetes-5^c^ ^23^	5	0–20	>7: elevated diabetes distress	Diabetes distress
Generalised Anxiety Disorder-7^d^ ^24^	7	0–21	0–4: minimal anxiety symptoms 5–9: mild anxiety symptoms 10–14: moderate anxiety symptoms ≥15: severe anxiety symptoms	Anxiety symptoms

^a^5-point Likert scale ranging from 1 (very good) to 5 (very bad). ^b^6-point Likert scale ranging from 0 (none of the time) to 5 (all of the time). ^c^5-point Likert scale ranging from 0 (not a problem) to 4 (serious problem). ^d^4-point Likert scale ranging from 0 (not at all) to 3 (nearly all of the time). WHO = World Health Organization.

Additionally, responders provided information on their education level and whether they lived alone.

### Data collection

The questionnaires were completed electronically or on paper, depending on the responder’s capability and preference. The recruiting healthcare professional registered the sex and age of all invited patients on the online registration webpage. The patients completed the questionnaires without involvement from healthcare professionals.

Reminders were sent to non-responders via email and text message after 1 and 2 weeks. The data were stored in an encrypted online database maintained by Aarhus University, and all identifiers were removed before data analysis.

### Statistical analyses

All analyses were performed using the statistical software package R (version 4.3.0).

We aimed for a sample of 230 responders to accurately estimate mean scores across various measures with a 95% confidence interval (CI) level. A descriptive analysis of the population was conducted to provide an overview of patient characteristics. Mean sum scores for the different PROMs were calculated for each sex and age group. Furthermore, a descriptive analysis of sex and age demographics was carried out for patients who either declined to participate or did not respond.

A Venn diagram was employed to illustrate the overlap between the different PROMs. Moreover, we compared patients with symptoms of impaired mental health, distinguishing between those identified by the WHO-5 questionnaire and those not identified by the WHO-5 questionnaire. A similar comparison was conducted for the single-item question on wellbeing.

All categorical values are presented as number (%) or median (interquartile range; IQR). Continuous variables are expressed as means (standard deviation). *T*-tests were employed for mean comparisons and presented with 95% CI.

## Results

### Mental health of patients with T2D in Danish general practice

A total of 369 patients were invited to complete the questionnaire; 98 declined to participate, and 41 did not complete the questionnaire, resulting in a final participation rate of 62%. Table 2 presents the characteristics of the responders and the prevalence of impaired mental health, according to different PROMs. A higher percentage of non-participants were male (prevalence: 68%) compared with the group of participants (prevalence: 60%). No significant differences were seen for age; the median age was 68 (IQR 57–76) years for non-participants and 66 (IQR 58–74) years for participants (data not shown).

**Table 2.** Characteristics of responders (*N* = 230)

Characteristic	*n* (%)
**Sex**	
Female	91 (40)
Male	139 (60)
**Age, years**	
0–39	7 (3)
40–59	63 (27)
60–79	136 (59)
≥80	24 (10)
**Education**	
Primary and lower secondary school	63 (27)
Upper secondary education	12 (5)
Vocational education	44 (19)
Short-cycle higher education	44 (19)
Medium-cycle higher education	65 (28)
Long-cycle higher education	2 (1)
**Civil status**	
Lives alone	63 (27)
**Mental health status**	
Subjective impaired wellbeing^b^	9 (4)
Impaired wellbeing^c^	39 (17)
Moderate-to-severe depressive symptoms^d^	52 (23)
Diabetes distress^e^	37 (16)
Moderate-to-severe anxiety symptoms^f^	18 (8)

^b^Single-item wellbeing question: score ≥4. ^c^World Health Organization-Five Wellbeing Index: score <50.^d^Patient Health Questionnaire-9: score ≥10. ^e^Problem Areas in Diabetes-5: score >7. ^f^Generalized Anxiety Disorder-7: score ≥10

Negligible differences were observed in the psychometric summary scores between males and females (Table 3). Younger patients exhibited significantly higher levels of impaired wellbeing and of anxiety symptoms.

**Table 3.** Patient-reported outcome measures stratified by sex and age

Patient-reported outcome measures	Female^a^	Male^a^	Mean difference^b^	Aged <66 years^a^	Aged ≥66 years^a^	*P*-value^b^
Single-item wellbeing question	2.05 (0.83)	1.92 (0.87)	0.13 (–0.09 to 0.36)	2.03 (0.87)	1.93 (0.84)	0.10 (–0.12 to 0.33)
WHO-5 Wellbeing Index	65.80 (19.49)	70.13 (19.64)	–4.33 (–9.53 to 0.87)	65.45 (21.42)	71.13 (17.53)	–5.68 (–10.79 to –0.56)
Patient Health Questionnaire-9	6.13 (4.94)	5.19 (4.99)	0.94 (–0.38 to 2.26)	6.12 (5.43)	5.06 (4.48)	1.06 (–0.24 to 2.36)
Problem Areas in Diabetes-5	4.16 (4.02)	3.37 (4.10)	0.79 (–0.29 to 1.87)	4.15 (4.26)	3.26 (3.87)	0.89 (–0.16 to 1.96)
Generalised Anxiety Disorder-7	3.22 (4.16)	2.76 (3.81)	0.46 (–0.61 to 1.54)	3.77 (4.72)	2.18 (2.89)	1.59 (0.57 to 2.63)

^a^Mean (standard deviation). ^b^Mean difference (95% confidence interval). WHO = World Health Organization.

### WHO-5 questionnaire and single-item question on wellbeing

**Figure 1.**
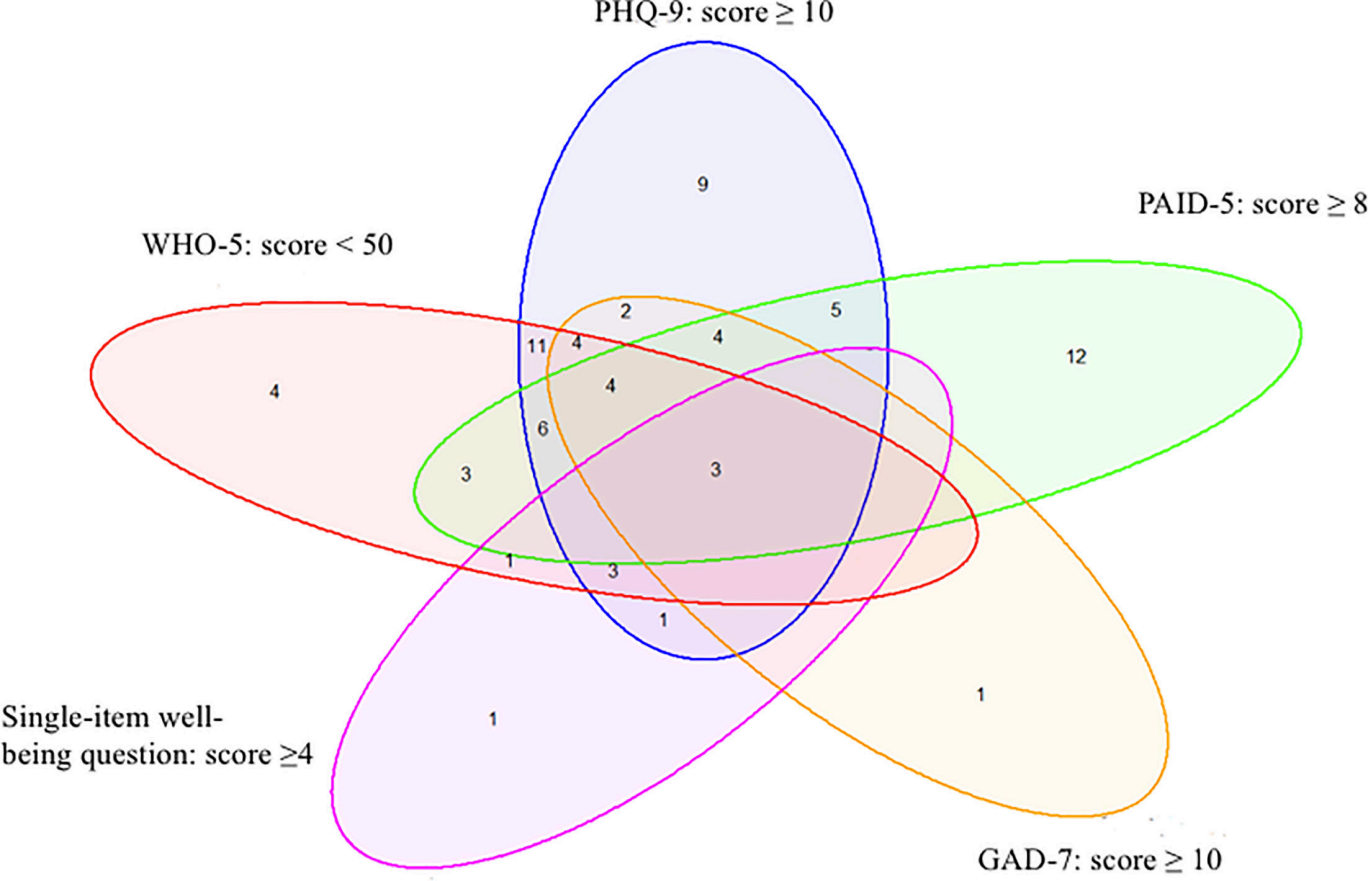
Venn diagram showing the number of people identified by the different patient-reported outcome measures. A total of 74 people exhibited impaired mental health. GAD-7 = Generalized Anxiety Disorder-7. PAID-5 = Problem Areas in Diabetes-5. PHQ-9 = Patient Health Questionnaire-9. WHO-5 = World Health Organization-Five Wellbeing Index.

Figure 1 is a Venn diagram illustrating the overlap and unique responses among the different PROMs. Out of the entire study population, 74 patients (32%) reported symptoms of impaired mental health on at least one of the PROMs. Specifically, the single-item wellbeing question identified 12% of these patients, the WHO-5 53%, the PHQ-9 70%, the PAID-5 50%, and the GAD-7 24%. The Venn diagram reveals that the WHO-5 had four unique responses and 35 responses that overlapped with ≥1 of the other PROMs. This suggests that the WHO-5 has a significant level of correlation with other measures of mental health. In contrast, the PAID-5 identified 12 unique responses and only 25 that overlapped with other PROMs, indicating a smaller degree of overlap.

The comparison of mean scores across the different PROMs in patients exhibiting symptoms of impaired mental health revealed that those identified via the single-item question had a slightly higher, but not statistically significant, degree of impaired mental health compared with those not identified. Similar trends were observed for the WHO-5 questionnaire, however, with all differences being statistically significant except for diabetes distress (Table 4). A similar comparison for the PAID-5 showed no statistically significant value differences for the other psychometric measures (see Supplementary Table S1). These findings, along with Figure 1, suggest that diabetes distress, measured by the PAID-5, does not correlate with the other PROMs. Conversely, wellbeing, measured by the WHO-5, correlates significantly with both anxiety and depressive symptoms.

**Table 4.** Mental health metrics in cases (*n* = 74)

Patient-reported outcome measures	Impaired single-item wellbeing, mean (SD)^a^	Good single-item wellbeing, mean (SD)^a^	Mean difference (95% CI)	Impaired WHO-5 wellbeing, mean (SD)^b^	Good WHO-5 wellbeing, mean (SD)^b^	Mean difference (95% CI)
Single-item wellbeing question	4.00 (0.00)	2.48 (0.71)	1.52 (1.35 to 1.70)	3.00 (0.65)	2.29 (0.86)	0.71 (0.36 to 1.07)
WHO-5^b^	36.00 (21.91)	51.32 (17.77)	–15.32 (–32.44 to 1.79)	34.67 (11.47)	65.94 (9.17)	–31.37 (–36.10 to –26.50)
PHQ-9^c^	14.56 (4.59)	10.55 (4.03)	4.01 (0.41 to 7.60)	12.46 (4.10)	9.46 (3.94)	3.00 (1.14 to 4.87)
PAID-5^d^	7.89 (6.64)	7.58 (4.06)	0.31 (–4.84 to 5.45)	6.85 (5.00)	8.49 (3.58)	–1.64 (–3.60 to 0.32)
GAD-7^e^	10.22 (6.76)	6.05 (4.34)	4.17 (–1.07 to 9.42)	7.87 (5.03)	5.09 (4.20)	2.78 (0.64 to 4.93)

^a^≥4 = impaired single-item wellbeing. ^b^WHO-5 Wellbeing Index, <50 = impaired WHO-5. GAD-7 = Generalised Anxiety Disorder-7. PAID-5 = Problem Areas in Diabetes-5. PHQ-9 = Patient Health Questionnaire-9. SD = standard deviation. WHO-5 = World Health Organization-Five Wellbeing Index.

## Discussion

### Summary

In this general practice T2D population, one in three patients reported symptoms of impaired mental health, as indicated by one or more of the used PROMs. The identified individuals mainly reported symptoms related to impaired wellbeing, depression, and diabetes distress.

The WHO-5 questionnaire demonstrated superior performance compared with the single-item question in identifying patients with symptoms of impaired mental health. The WHO-5 identified impaired mental health in 17% of responders, while the single-item wellbeing question identified only 4%. These findings suggest that the prevalence of impaired mental health could be underestimated by relying solely on direct questioning. While the WHO-5 questionnaire may not capture every patient exhibiting symptoms of impaired mental health, our findings suggest that it excels in identifying those with the most pronounced impairments. Consequently, it holds significant potential as a systematic tool for identifying patients with impaired mental health. This potential is reinforced by the brevity of WHO-5 and its positively formulated questions.

Numerous studies have examined the mental health of patients with T2D, but this study is, to our knowledge, the first to conduct a nuanced investigation exclusively in a general practice setting. Our findings provide novel insights into the mental health of this population, emphasising the importance of recognising mental health within the context of general practice. Furthermore, the study offers novel perspectives on the effectiveness of employing a psychometric measure, compared with single-item questioning, to identify impaired mental health in individuals with T2D in general practice.

### Strengths and limitations

Our study has several important strengths. It exclusively involves the population of interest: patients with T2D treated in general practice. Knowledge about this specific population is essential for the clinician managing patients with T2D in general practice. The patients were provided with the option to respond either electronically or on paper, thereby facilitating participation for those with restricted electronic access or limited ability to respond digitally. This approach broadens the generalisability of our results. Our participation rate of 62% is higher than would typically be anticipated in an online questionnaire survey.^26^ This is most likely owing to personal recruitment by the healthcare professionals. Furthermore, no significant differences were seen in the age and sex composition between responders and non-responders, suggesting generalisable results.

The study also has some limitations, for example, the relatively small sample size of the population. The variation in participation rates across different clinics could be owing to different demographics or variations in recruitment strategies by the healthcare professionals. Additionally, although the healthcare professionals were instructed to recruit patients consecutively, not all eligible patients may have been recruited owing to factors such as time constraints or oversight by healthcare professionals, which might have introduced a risk of recruitment bias. Importantly, patients excluded owing to language barriers may represent an even more vulnerable subpopulation regarding mental health. This study provides no information on this specific group.

Although we made a preliminary assessment of the single-item wellbeing question, no formal validation procedures were applied. The single-item wellbeing question was used to simulate inquiries that healthcare professionals would make in a clinical setting. However, replicating such a question in writing poses challenges, as factors such as tone, body language, setting, and the professional relationship between the patient and the healthcare professional cannot be conveyed. Although the single-item question might offer an indication of how patients are likely to respond to this type of inquiry in a clinical setting, additional studies are needed to assess its validity.

### Comparison with existing literature

In this study, 32% of responders reported symptoms of impaired mental health on at least one of the PROMs, with the majority experiencing impaired wellbeing, depressive symptoms, and diabetes distress. These findings are comparable with those in other studies, where depression rates were found to be between 19% and 31%,^2–5^ In another study, impaired psychological wellbeing was found to be 40%.^27^ We found that younger patients had significantly higher levels of impaired wellbeing and anxiety symptoms. This aligns with findings from previous studies,^28,29^ and highlights the need for early and proactive attention to mental health in diabetes care.

This study suggests that clinicians may fail to identify significant mental health problems if the only assessment method used is simply asking patients how they are. Clinicians may resort to asking this rather simple question during a busy consultation, where numerous tasks need attention. Consequently, they may not gain a complete understanding of the patient’s true wellbeing. This corresponds well with previous research, which has indicated that depression in individuals with T2D is often undiagnosed, despite regular interactions with their clinician.^30,31^ Patients with T2D typically do not expect mental health to be addressed during a diabetes consultation, which may contribute to a higher number of undiagnosed cases of depression.^32,33^ A strong relationship between the healthcare professional and the patient means that the patient is more likely to discuss their mental health. When this relationship is not established, using a PROM might be even more valuable.^34^ From the healthcare professionals’ perspective, some of the barriers may be reluctance to open 'a can of worms', feeling insufficiently trained to deal with possible depression, and lack of knowledge on guidelines.^32,35^ These barriers are important considerations in discussing the potential implementation of systematic mental health assessments in patients with T2D.

Previous studies have established the WHO-5 questionnaire as a sensitive and specific screening tool for depression.^19^ Our study reinforces this finding. While it may not identify all patients experiencing symptoms of impaired mental health, our findings indicate that it succeeds in identifying the patients with the most severe symptoms of depression and anxiety.

While using a diabetes-specific questionnaire, such as the PAID-5, in a population with diabetes might be tempting, our study found no statistically significant differences in the other psychometric measures between patients showing symptoms of impaired mental health identified by PAID-5 and patients expressing similar symptoms that were not captured by the PAID-5. This suggests that the PAID-5 may be less effective than the WHO-5 questionnaire for a comprehensive assessment of mental health, given that its original purpose is to measure diabetes distress rather than general mental health.

Evidently, not all patients meeting the criteria for impaired wellbeing, according to the WHO-5 questionnaire, will experience depression or another form of impaired mental health. This situation can result in some false positives, which holds a risk of overdiagnosis. Meeting the criteria may place emotional strain on the patient, but it may also require the GP to invest additional time in the patient, thereby contributing to escalated costs and increased workloads. It is crucial to emphasise that the WHO-5 questionnaire is not designed as a diagnostic tool. Instead, it should be employed as a screening instrument, and results must be followed-up by a clinical evaluation. Additionally, in the pursuit of identifying more patients with impaired mental health, it is crucial to ensure the ready availability of effective treatments such as talking therapy or antidepressants.^36^

### Implications for research and practice

This study shows that impaired mental health is common in patients with T2D in general practice, emphasising the importance of addressing mental health in this population in this setting. However, when implementing a systematic assessment, it is crucial to ensure that a well-defined system for managing the outcomes is established. This necessitates educating healthcare professionals in effectively handling mental health concerns, implementing clear guidelines for the management of such patients, and, importantly, ensuring allocation of adequate time for discussions about mental health during consultations.

Most likely, implementing a systematic mental health assessment will lead to increased healthcare costs in the short run, as more patients will be identified with impaired mental health and need for treatment. However, improving mental health in these patients may result in better somatic health outcomes, higher quality of life, and fewer interactions with the healthcare system, thereby reducing costs in the long run. This needs to be investigated in future studies.

Our study suggests that the WHO-5 questionnaire could be a useful screening tool to identify significant mental health problems that may otherwise go unrecognised. The WHO-5 questionnaire is a brief, positively phrased questionnaire, which may be more welcomed by patients than the negatively phrased questions in, for example, the PHQ-9. Further studies are needed to assess the suitability of the WHO-5 questionnaire in a primary care setting. This includes qualitative studies to capture the perspectives of both patients and healthcare workers regarding its use.

In conclusion, this study provides evidence that impaired mental health affects a substantial proportion of patients with T2D treated in general practice. Furthermore, our findings suggest that mental health issues are less likely to be identified when patients are questioned about their mental wellbeing with a single-item question compared with assessment with the WHO-5 questionnaire. To ensure a comprehensive approach to addressing mental health in these patients, systematic assessment with the WHO-5 questionnaire may be valuable.

## Data

All data behind the conclusions in this article are viewable in the tables provided.
